# First-in-human phase I study of EMB-02, a bispecific antibody targeting PD-1 and LAG-3 in patients with advanced solid tumors

**DOI:** 10.1038/s41416-025-02990-x

**Published:** 2025-04-15

**Authors:** Daphne Day, Vinod Ganju, Ki Chung, Lu Si, Lili Mao, Morteza Aghmesheh, Robert Hoyer, Kim Brewin, Shuqi Zeng, Mingfei Zhang, Qiaoyang Lu, Chengjun Jiang, Fang Ren, Yonghong Zhu, Jun Guo

**Affiliations:** 1 Medical Oncology Department, Monash Health—Monash MedicalCentre, Clayton, VIC Australia; 2 Oncology Department, Peninsula And Southeast Oncology, Frankston, VIC Australia; 3 Department of Medicine, Prisma Health Cancer Institute, Greenville, SC USA; 4 Key Laboratory of Carcinogenesis and Translational Research (Ministry of Education/Beijing), Department of Renal Cancer and Melanoma, Collaborative Innovation Center for Cancer Medicine, Peking UniversityCancer Hospital and Institute, Beijing, China; 5 Medical Oncology Department, Prince of Wales Hospital, Sydney, NSW Australia; 6 Medical Oncology Department, UCHealth Memorial Hospital Central, Colorado Springs, CO USA; 7 Clinical Development, Shanghai EpimAb Biotherapeutics Co., Ltd., Shanghai, China

**Keywords:** Drug development, Cancer immunotherapy

## Abstract

**Background:**

EMB-02 is a symmetric bispecific antibody targeting programmed cell death protein-1 and lymphocyte-activation gene 3 simultaneously. Here, we present the first-in-human study results of EMB-02 in patients with advanced solid tumors.

**Methods:**

Patients were treated with intravenous infusions of EMB-02 at doses of 6–900 mg. The primary objective was to evaluate the safety and tolerability and to determine the maximum tolerated dose and/or recommended phase II dose(s). Secondary objectives included characterizing the pharmacokinetic (PK) profile, assessing preliminary antitumor activity and the immunogenicity.

**Results:**

A total of 47 patients were enrolled. All grade and grade 3/4 treatment-emergent and treatment related adverse events occurred in 97.9%, 48.9%, 68.1% and 12.8% patients, respectively. The objective response rate (ORR) was 6.4% and clinical benefit rate at 24 weeks (CBR-24) was 25.5% in overall population. The CBR-24 was 33.3% in checkpoint inhibitor (CPI)-naïve patients, and 15% in CPI-treated. No clear relationship was observed between the efficacy and PD-L1, LAG-3, or MHC II expression level. Doses 360 mg or higher resulted in sustained saturation of PD-1 receptors on circulating CD3 + T cells.

**Conclusions:**

EMB-02 demonstrated a favorable safety profile and early efficacy signals in multiple solid tumors, warranting further development. (NCT04618393).

## Introduction

In 2014, the first anti- programmed death-1 (PD-1) monoclonal antibody was approved by the FDA for the treatment of melanoma, marking a significant milestone in immunotherapy. Over the next decade, multiple agents targeting PD-1 or programmed death-ligand 1 (PD-L1) have been approved to treat various tumors. However, PD-1/PD-L1 CPIs, have only shown significant clinical benefits in a subset of patients. Furthermore, secondary resistance and undesired immunogenicity can arise in patients who derive initial benefit [1, 2]. Hence, it is necessary to explore additional strategies, such as those targeting the tumor microenvironment to restore effector T cell function and overcome drug resistance.

Lymphocyte activation gene-3 (LAG-3) is a transmembrane protein on activated T cells, acting as a co-inhibitory receptor that suppresses T cell functions [3]. LAG-3 interacts with ligands such as major histocompatibility complex II (MHC-II) expressed by tumor or innate immune cells, and fibrinogen-like protein 1 (FGL-1), highly expressed in some tumors [4]. Galectin-3 and LSECtin also interact with LAG-3, but their roles in T cell suppression are less understood [5]. Like PD-1, LAG-3 is an “exhaustion” marker of CD8 + T cells, indicating dysfunctional T cells from chronic stimulation, as seen in animal models of chronic infection and cancer [6–8]. Additionally, LAG-3 is expressed by regulatory T cells in the tumor microenvironment, contributing to their suppressive activities [9]. In numerous nonclinical models, blocking both LAG-3 and PD-1 improved T cell function, aiding in viral and parasitic infection clearance, and enhancing CD8 + T cell-mediated anti-tumor activity [7, 10]. Overexpression of LAG-3 and/or PD-1 has been observed in many human cancers, including melanoma, non-small cell lung cancer (NSCLC), colorectal cancer (CRC), breast cancer, and Hodgkin lymphoma [11–13]. Therefore, combined blockade of LAG-3 and PD-1 might more efficiently overcome T-cell exhaustion mechanisms and restore effector functions, leading to tumor regressions [14–16].

The combination of nivolumab and relatlimab (anti-LAG-3 antibody) showed significantly higher response rate and progression-free survival compared to nivolumab alone and has been approved by the FDA for the treatment of unresectable or metastatic melanoma [17]. Other co-blockade treatments either using combined monoclonal antibodies or bispecific antibodies have also demonstrated preliminary efficacy in both solid tumors and hematological malignancies, such as NSCLC, microsatellite stable (MSS) CRC, epithelial ovarian cancer, triple negative breast cancer (TNBC), diffuse large B-cell lymphoma and relapsed or refractory classical Hodgkin lymphoma in clinical trials [18–21].

EMB-02 is a symmetric tetravalent IgG-like bispecific antibody against PD-1 and LAG-3 developed by Fabs-In-Tandem Immunoglobulin (FIT-Ig) platform, designed to target human PD-1 and LAG-3 concomitantly or independently to disrupt the immune suppression mediated by both pathways, thereby restoring T-cell effector function to enhance anti-cancer immunity. To reduce potential effector-function induced depletion of immune cells expressing PD-1 or LAG-3, the human IgG1 Fc domain of EMB-02 was engineered to contain a LALA double mutation (mutation of leucine residues at positions 234 and 235 into alanine residues) [22]. Here, we report the dose escalation results of the first-in-human (FIH) study of EMB-02, in patients with advanced solid tumors (ClinicalTrials. gov identifier: NCT04618393).

## Methods

### Study design and treatment

This study was a phase I/II, open-label, dose escalation study conducted in six sites in Australia, China and USA. The primary objectives of the phase I portion were to evaluate the safety and tolerability of EMB-02 and to determine the MTD and/or RP2D. The secondary objectives included assessing the PK profile, preliminary antitumor activity, and immunogenicity. The preliminary evaluation of EMB-02 antitumor activity included best overall response (BOR), objective response rate (ORR), disease control rate (DCR), clinical benefit rate at 24 weeks (CBR-24, which is defined as the proportion of patients with a complete or partial response or remain stable disease at Week 24 assessment), duration of response (DOR) and progression free survival (PFS) based on modified Response Evaluation Criteria in Solid Tumors Version 1.1 (RECIST V1.1) criteria. In this study, durable clinical benefit is defined as achieving clinical benefit at 24 weeks.

The phase I portion of this study consisted of two stages. Stage 1 involved dose escalation to explore the safety and tolerability of EMB-02 at dose levels of 6 mg, 20 mg, 60 mg, 180 mg, 360 mg, 600 mg and 900 mg via intravenous infusion, once weekly. Stage 2 involved dose enrichment, where additional patients were added at the identified potential efficacious dose levels of 60 mg, 180 mg and 600 mg. The starting dose of 6 mg of EMB-02, derived using the minimal anticipated biological effect level (MABEL) approach. Dose escalation was guided by BOIN design with a target DLT rate of 25%, including an initial Accelerated Titration Design stage. Intra-patient dose increases were allowed after at least two treatment cycles if, in the opinion of the treating investigator and the sponsor, a patient who had received a lower dose which he/she was initially assigned may benefit from a higher dose that has been shown to be safe and tolerated during dose escalation (not exceeding MTD). After evaluating the initial PK data, the dosing interval might be increased (e.g. to 2-weekly) if drug accumulation occurred upon repeated dosing. Patients remained on EMB-02 treatment until confirmed disease progression, death, intolerable toxicity, withdrawal of consent, investigator decision, or any other treatment discontinuation criteria.

The infusion time of EMB-02 was no less than 60 min, no more than 120 min, and the interval between doses in the once-weekly regimen should be no less than 5 days. Diphenhydramine (25 or 50 mg) intravenously or intramuscularly 30–60 min prior to EMB-02 dosing in the first two cycles was recommended. Other optional pre-medications included dexamethasone 10 mg IV or prednisone/equivalent 0.5 mg/kg (maximum dose of 50 mg) PO and/or oral acetaminophen. Premedication for subsequent treatment cycles depended on whether the patient developed infusion-related reactions (IRR) or at the discretion of the investigator. The study complied with international standards of Good Clinical Practice and the Declaration of Helsinki and all applicable regulatory requirements. The protocol was approved by the institutional review boards or ethics committees. All patients provided written informed consent before study enrollment.

### Patient population

Eligible patients were aged ≥18 years with an Eastern Cooperative Oncology Group performance status (ECOG PS) of 0 or 1. Patients had histologically or cytologically confirmed locally advanced or metastatic solid tumors, have failed (progressed on or after or were intolerant to) standard therapies and had measurable or evaluable disease per RECIST v1.1. To be enrolled, patients were required to provide archival tumor samples or a fresh biopsy if archival tumor sample was unavailable. Eligible patients also needed to have adequate organ function without significant adverse events (AEs) related to prior treatment(s).

Key exclusion criteria included prior treatment with any anti-LAG-3 therapy, symptomatic central nervous system metastases, active or history of autoimmune disease, history of Grade 3 or 4 immune-related adverse events (irAEs) or irAEs requiring discontinuation of prior therapies, use of high dose systemic corticosteroids, clinically significant cardiovascular disease, active infection, and/or any other serious underlying medical conditions.

### Study assessments

Safety measurements were conducted at each visit including clinical laboratory tests, physical examinations, vital signs, ECOG PS and electrocardiograms (ECGs). The dose-limiting toxicity (DLT) observation period was 28 days. Unless a DLT was observed during the DLT observation period, DLT-evaluable patients must have received at least 75% of planned doses and the DLT evaluability of each patient was discussed and confirmed at Safety Review Committee meeting. AEs, including treatment-emergent AEs (TEAEs), treatment-related AEs (TRAEs), irAEs and serious AEs (SAEs) classified by the National Cancer Institute Common Terminology Criteria for Adverse Events (CTCAE V5.0), were assessed at each visit. AEs of special interest (AESI) encompassed irAEs and infusion related reactions (IRRs) in this study.

Efficacy evaluations were performed by investigators according to modified RECIST V1.1. Computed tomography (CT) or magnetic resonance imaging scans were conducted at baseline and then repeated every 8 weeks ±7 days for the first 48 weeks and every 12 weeks ±7 days thereafter. Radiologic progression without clinically significant deterioration required confirmation with a subsequent scan, and the confirmatory scan should be performed no less than 4 weeks after the prior assessment of progression to exclude pseudoprogression.

Blood samples were collected at specific time points to determine the serum concentrations of EMB-02 using a validated immunoassay for PK analysis. PK parameters of EMB-02 were determined using non-compartment methods. Additionally, blood samples were collected to assess PD-1 receptor occupancy on circulating CD3 + T cells by a flow cytometry assay for pharmacodynamic (PD) analysis, and to test the incidence and titer of anti-drug antibodies (ADAs) to EMB-02 for immunogenicity evaluation.

Archived formalin-fixed, paraffin-embedded tumor specimens or fresh tumor biopsies collected prior to EMB-02 treatment were analyzed for PD-L1, LAG-3, and MHC II expression. Immunohistochemical staining was performed using antibody clones SP263 (Ventana) for PD-L1, CAL26 (Biocare Medical) for LAG-3, and LGII-612.14 (Cell Signaling Technology) for MHC II.

### Statistical analysis

Descriptive statistics were provided for selected demographics and baseline characteristics, efficacy, safety, PK/ADA, pharmacodynamics, and biomarker data. The Clopper-Pearson method was used to calculate the 95% confidence interval of ORR, DCR and CBR-24. The Kaplan-Meier method was used to estimate the median and 95% confidence interval of DOR and PFS if data permitted.

## Results

### Patient disposition and baseline characteristics

**Fig. 1:**
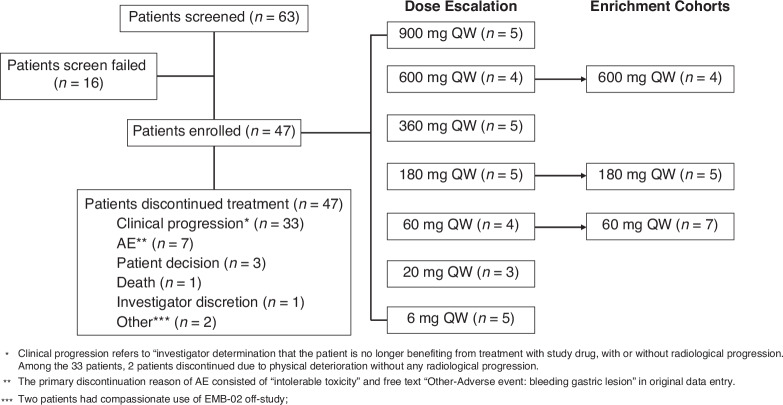
Patient flow diagram.

Between February 1, 2021 and July 12, 2023, 47 patients with advanced/metastatic solid tumors were enrolled. At study completion, all 47 (100%) patients had discontinued treatment. The most common primary reasons for treatment discontinuation were clinical progression (*n* = 33 [70.2%]) and AEs (*n* = 7 [14.9%]) (Fig. 1). The median duration of exposure was 12-weeks (interquartile range [IQR], 7.3–29.1) and the median number of EMB-02 doses received was 12 (IQR, 6–24).

Demographics and baseline characteristics are summarized in Table 1. Overall, 19 patients (40.4%) were male, with a median age of 61 years (range: 37–83 years inclusive). The most common tumor type was melanoma (*n* = 12, 25.5%; acral melonma, *n* = 5; mucosal melanoma; *n* = 3; cutaneous melanoma, *n* = 2; uveal melanoma, *n* = 1; unknown subtype, *n* = 1). Other common tumor types included colorectal cancer (*n* = 10, 21.3%), all of which were MSS, and non-small cell lung cancer (NSCLC, *n* = 4, 8.5%). Twenty (42.6%) patients had received a prior CPI, and 21 (44.7%) patients had received ≥3 prior lines of treatment.

**Table 1:** Baseline characteristics.

Demographics and baseline characteristics	Overall patients (*N* = 47)
Median age (range), years	61 (37–83)
Female; n(%)	28 (59.6)
Race [n (%)]	
Black or African American	2 (4.3)
Asian	10 (21.3)
Native Hawaiian or Other Pacific Islander	1 (2.1)
White	34 (72.3)
ECOG PS; n(%)	
0	31 (66)
1	16 (34)
Median Time Since Initial Diagnosis(range), years	2.11 (0.3–14.4)
Tumor type; n(%)	
Melanoma	12 (25.5)
Colorectal cancer	10 (21.3)
Non-small cell lung cancer	4 (8.5)
Triple negative breast cancer	3 (6.4)
Esophageal squamous cell carcinoma	3 (6.4)
Gastric cancer	3 (6.4)
Other	12 (25.5)
Prior CPI therapy; n (%)	20 (42.6)
anti-PD-(L)1 only	18 (38.3)
anti-PD-(L)1+anti-CTLA-4	2 (4.3)
Prior cancer-related surgery; n (%)	41 (87.2)
Prior radiotherapy; n (%)	32 (68.1)
Prior lines of systemic therapy; n (%)	
0	1 (2.1)
1–2	25 (53.2)
≥3	21 (44.7)

### Safety and tolerability

All 47 treated patients were included in the safety analysis. The MTD of EMB-02 was not reached according to the BOIN design, as only one DLT event (Grade 4 immune-mediated hepatitis) was identified at 900 mg of five patients. Dose escalation was concluded at this level. A safety overview is summarized in Table 2. Forty-six of 47 patients (97.9%) experienced at least one TEAE of any grade (Supplementary Table S1). The most common (≥20%) TEAEs were fatigue and nausea (*n* = 13, 27.7% each), IRRs and vomiting (*n* = 11, 23.4% each), and constipation (*n* = 10, 21.3%).

**Table 2:** Safety overview.

Safety overview	All patients (*N* = 47) N (%)
Patients with TEAE	46 (97.9)
Patients with treatment-related AE	32 (68.1)
Patients with SAE	17 (36.2)
Patients with treatment-related SAE	5 (10.6)
Patients with Grade 3 or above AE	23 (48.9)
Patients with Grade 3 or above treatment related AE	6 (12.8)
Patients with AE leading to treatment discontinuation	10 (21.3)
Patients with treatment related AE leading to treatment discontinuation	5 (10.6)
Patients with AE leading to death	4 (8.5)
Patients with treatment related AE leading to death	0
Patients with DLT	1 (2.1)

Thirty-two (68.1%) patients experienced TRAEs (Table 3). The most commonly reported (≥10%) TRAEs were IRRs (*n* = 11, 23.4%), fatigue (*n* = 7, 14.9%) and diarrhea (*n* = 5, 10.6%). Five patients (10.6%) experienced TRAEs leading to treatment discontinuation, including immune-mediated hepatitis (*n* = 3, 6.4%), hypertransaminasaemia, ALT increased, AST increased, increased blood bilirubin, IRRs and lung consolidation (*n* = 1, 2.1% each). There was no TRAE leading to death. No clear dose-toxicity relationship was found from the perspective of TRAE rates, however EMB-02 exhibited relatively higher liver toxicities at higher doses.

**Table 3:** Treatment related adverse events (TRAEs) with an incidence of ≥ 5% in the safety population.

	6 mg QW (*N* = 5)		20 mg QW (*N* = 3)		60 mg QW (*N* = 11)		180 mg QW (*N* = 10)		360 mg QW (*N* = 5)		600 mg QW (*N* = 8)		900 mg QW (*N* = 5)			Total (*N* = 47)	
Preferred Term	Any grade n (%)	Grade ≥ 3 n (%)	Any grade n (%)	Grade ≥ 3 n (%)	Any grade n (%)	Grade ≥ 3 n (%)	Any grade n (%)	Grade ≥ 3 n (%)	Any grade n (%)	Grade ≥ 3 n (%)	Any grade n (%)	Grade ≥ 3 n (%)	Any grade n (%)	Grade ≥ 3 n (%)	Any grade n (%)		Grade ≥ 3 n (%)
**Subjects with Any Drug-related AE (%)**	3 (60.0)	0	3 (100)	0	7 (63.6)	1 (9.1)	6 (60.0)	1 (10.0)	3 (60.0)	0	7 (87.5)	2 (25.0)	3 (60.0)	2 (40.0)	32(68.1)		6 (12.8)
**Infusion related reaction**	1 (20.0)	0	2 (66.7)	0	2 (18.2)	1 (9.1)	2 (20.0)	0	0	0	3 (37.5)	1 (12.5)	1 (20.0)	0	11(23.4)		2 (4.3)
**Fatigue**	1 (20.0)	0	0	0	3 (27.3)	0	1 (10.0)	0	0	0	1 (12.5)	0	1 (20.0)	0	7 (14.9)		0
**Diarrhea**	2 (40.0)	0	0	0	2 (18.2)	0	1 (10.0)	0	0	0	0	0	0	0	5 (10.6)		0
**GGT increased**	0	0	0	0	0	0	0	0	1 (20.0)	0	2 (25.0)	1 (12.5)	1 (20.0)	1 (20.0)	4 (8.5)		2 (4.3)
**ALT increased**	0	0	0	0	0	0	0	0	0	0	2 (25.0)	1 (12.5)	1 (20.0)	1 (20.0)	3 (6.4)		2 (4.3)
**Arthralgia**	0	0	0	0	0	0	2 (20.0)	0	0	0	0	0	1 (20.0)	0	3 (6.4)		0
**AST increased**	0	0	0	0	0	0	0	0	0	0	2 (25.0)	1 (12.5)	1 (20.0)	1 (20.0)	3 (6.4)		2 (4.3)
**Blood bilirubin increased**	0	0	0	0	0	0	1 (10.0)	0	0	0	1 (12.5)	0	1 (20.0)	1 (20.0)	3 (6.4)		1 (2.1)
**Hypothyroidism**	0	0	0	0	0	0	2 (20.0)	0	0	0	1 (12.5)	0	0	0	3 (6.4)		0
**Immune-mediated hepatitis**	0	0	0	0	0	0	1 (10.0)	1 (10.0)	0	0	0	0	2 (40.0)	1 (10.0)	3 (6.4)		2 (4.3)
**Lipase increased**	0	0	0	0	1 (9.1)	1 (9.1)	0	0	1 (20.0)	1 (20.0)	0	0	1 (20.0)	1 (20.0)	3 (6.4)		3 (6.4)
**Nausea**	0	0	1 (33.3)	0	1 (9.1)	0	0	0	0	0	1 (12.5)	0	0	0	3 (6.4)		0
**Pruritus**	0	0	0	0	0	0	1 (10.0)	0	0	0	2 (25.0)	0	0	0	3 (6.4)		0
**Pyrexia**	0	0	1 (33.3)	0	0	0	1 (10.0)	0	0	0	1 (12.5)	0	0	0	3 (6.4)		0
**Vomiting**	1 (20.0)	0	0	0	1 (9.1)	0	1 (10.0)	0	0	0	0	0	0	0	3 (6.4)		0

*GGT* Gamma-glutamyltransferase, *ALT* alanine aminotransferase, *AST* aspartate aminotransferase.

Fourteen patients (29.8%) reported irAEs of any grade, while 5 patients (10.6%) experienced grade 3 or 4 irAEs. The median time to first onset of any irAE was 27.5 (range: 1–165) days. IrAEs reported by at least two patients are summarized in Supplementary Table S2. Hepatic irAEs were a particular concern in this study, which were seen in four patients; all four patients had grade 3/4 events, including one DLT. The more commonly reported (≥2 patients) hepatic irAEs of any gade were immune-mediated hepatitis (*n* = 3, 6.4%), AST increased, ALT increased and GGT increased (*n* = 2, 4.3% each). The patient with reported DLT was found to have grade 4 immune-mediated hepatitis and grade 3 increases in AST, ALT, and blood bilirubin levels during cycle 1 of 900 mg QW treatment. Consequently, the patient withdrew from EMB-02 treatment and was treated with corticosteroids. The patient was recovering at the time of death, which was due to gastrointestinal bleeding unrelated to EMB-02 treatment. Other cases of high grade hepatotoxicity included: grade 2 immune-mediated hepatitis and grade 3 increase in GGT, 900 mg QW, cycle 1; grade 3 immune-mediated hepatitis, 180 mg QW, cycle 3; grade 3 increase in GGT and ALT, and grade 2 increase in AST, 600 mg QW, cycle 3. All three patients recovered after corticosteroid therapy with one patient requiring the addition of mycophenolate mofetil and all discontinued EMB-02 due to the hepatoxicity. For non-hepatic irAEs, the commonly reported (≥2 patients) irAEs of any grade were hypothyroidism, pruritus (*n* = 3, 6.4% each), and hyperthyroidism, lipase increased, pyrexia, arthralgia (*n* = 2, 4.3% each). With the exception of two patients with Grade≥3 lipase increase, all events were grade 1 to 2. The incidence of irAEs may correlate with dose level, as relatively higher incidence and severity were observed in patients treated at 900 mg, thus dose escalation was concluded at this level.

Eleven (23.4%) patients reported IRRs and these occurred at all dose levels; all cases were Grade 1 or 2, except two patients who had Grade 3 IRRs. The median time to first onset of IRRs was 15 (range: 1–170) days, which means the first IRRs usually occurred with the third dose. The most commonly reported symptoms were chills (*n* = 7, 14.9%), nausea (*n* = 4, 8.5%), back pain, and pruritus (*n* = 3, 6.4% each, Table S3). Treatment strategies included dose interruption, and/or supportive measures such as antihistamines, corticosteroids and paracetamol. In majority of cases, symptoms resolved within 24 h. Pre-medication at subsequent dosing mitigated further IRRs in most cases. Only one patient (60 mg QW) withdrew from treatment after experiencing a second episode of IRR. Five patients received corticosteroids for IRR/hypersensitivity prophylaxis, four of which were premedicated for recurrent IRR/hypersensitivity, while the remaining patient did not report any such event. Of the four premedicated patients with recurrent IRR/hypersensitivity, three tolerated the study drug well after steroid withdrawal, whereas one patient continued corticosteroid use for both IRR management and prophylaxis until disease progression.

### Efficacy

**Fig. 2:**
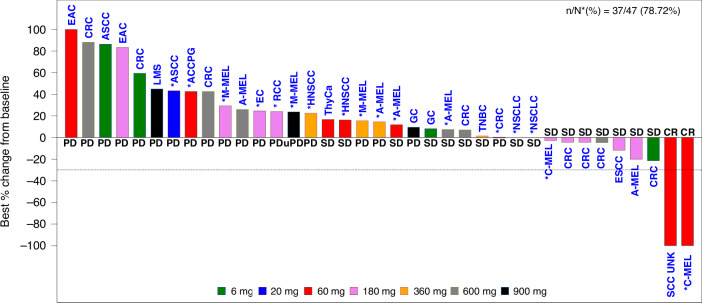
The efficacy of EMB-02 was demonstrated using a waterfall plot, which illustrates the percentage change from baseline in the sum of diameters of target lesions. The plot included 37 patients. Five patients were excluded due to the absence of measurable target lesions at baseline, and another five were excluded due to the lack of post-baseline radiological target lesion assessments. One ESCC patient with a BOR of CR was not represented in the plot due to the absence of a target lesion at baseline. Subjects who received prior treatment with an anti-PD-1/L1 inhibitor are indicated with an asterisk (*). For ten CRC patients, all of them were MSS. ACCPG Adenoid Cystic Carcinoma of the parotid gland, ASCC Anal squamous cell carcinoma (ASCC), CRC colorectal cancer, EAC esophageal adenocarcinoma, EC endometrial carcinoma, ESCC esophageal squamous cell carcinoma (ESCC), GC gastric cancer, HNSCC head and neck squamous cell carcinoma, LMS leiomyosarcoma, C-MEL cutaneous melanoma, A-MEL acral melanoma, M-MEL mucosal melanoma, NSCLC non-small cell lung cancer, RCC renal cell carcinoma, SCC UNK metastatic SCC of unknown primary, TNBC triple negative breast cancer, ThyCa Thyroid Carcinoma.

All 47 patients were included in efficacy analysis. ORR was 6.4% (95% CI, 1.34–17.54) in overall population. Eighteen (38.3%) patients achieved stable disease (SD), with seven (14.9%) patients having a reduction in overall tumor burden. DCR was 44.7% (95% CI, 30.17–59.88) and the CBR-24 was 25.5% (95% CI, 13.94–40.35) in overall population. The CBR-24 was 33.3% (9/27) in CPI naïve patients and 15% (3/20) in CPI pre-treated patients (Fig. 2). The overall PFS rate was 31.4% (95% CI, 18.18, 45.42) at 24 weeks and the overall median PFS was 8.0 weeks (95% CI, 7.4–15.9). No dose-related trend was observed.

Three patients obtained confirmed complete response (CR). These included two CPI naïve patients (ESCC, 6 mg QW switched to 12 mg Q2W after 15 weeks treatment, DOR 75.7 weeks; and squamous cell carcinoma of unknown primary, 60 mg QW, DOR 23.3 weeks and continues on EMB-02 treatment off-study) The third patient with non-acral cutaneous melanoma, BRAF V600 wild type, had a total exposure duration of 42.9 weeks, DOR of 72.4 weeks and remains in CR at the time of study closure. This Caucasian patient in their 60 s underwent curative surgery twice and received first-line nivolumab, second-line combined investigational anti-PD-1 and anti-CTLA-4 therapies before study entry. Target lesion was a lung metatasis measuring 17mm at baseline, patient achieved PR after 8 weeks of treatment and response deepened to CR with further dosing. Unfortunately the patient discontinued treatment due to immune-mediated bilateral peribronchovascular consolidation in lungs, which was also managed with corticosteroids.

Besides above mentioned cutaneous melanoma patient, another patient with cutaneous melanoma, also CPI-experienced, maintained stable disease for 24 weeks. Two acral melanoma patients with prior CPI treatment had SD at the week 8 assessment, and one CPI-naïve patient had a 20.0% reduction in target lesion at the week 16 assessment, although a new lesion was found at week 24 assessment. However, for patients with mucosal (*n* = 3) and uveal melanoma (*n* = 1), disease control was not achieved in any patient treated with EMB-02. No responses were observed in ten patients with MSS colorectal cancer, although four patients achieved durable SD with PFS ranging from 30.5 to 59.4 weeks. Notably, reduction in tumor size (21.4%) was seen in one CPI-naïve patient (KRAS G12D mutation positive, PD-L1 CPS ≥ 1) treated at 6 mg QW with disease control lasting 59.4 weeks before progression. Among the four patients with NSCLC, all of whom were CPI-exposed, no responses were observed, but one patient achieved durable SD with a PFS of 23.3 weeks. For the three CPI-naïve patients with TNBC, one patient in the 360 mg cohort achieved durable SD with a PFS of 53.3 weeks. Of the other two patients with ESCC (both CPI-naïve),both achieved durable SD for over 82 and 24.7 weeks, respectively. Of the three CPI-naïve patients with gastric cancer, one in the 6 mg cohort achieved durable SD with a PFS of 29.6 weeks.

### PK, immunogenicity, PD and biomarker analysis

**Fig. 3:**
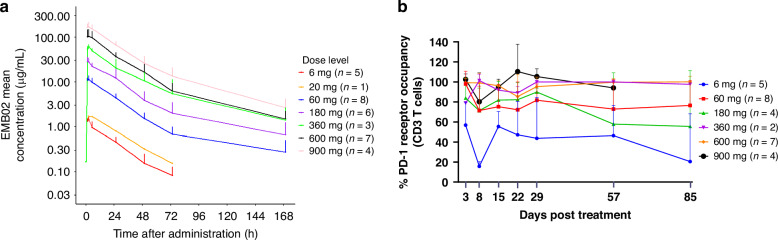
PK and PD profile of EMB-02. **a** The mean serum EMB-02 concentration following a single intravenous infusion at the tested dose levels (Semilogarithmic scale). **b** PD-1 receptor occupancy by EMB-02 on CD3 + T cells among patients treated at various doses. Data represent the mean + SD for each cohort. Notes: thirteen patients who had anti-PD-(L)1 treatment within four months of starting the study treatment and had higher pre-dose concentration were excluded from the PK concentration summary. Additionally, the PD-1 receptor occupancy data for seventeen patients were excluded from the analysis due to various reasons, including missing samples, extremely low levels of PD-1 expression, and prior treatment with other anti-PD-1 drugs within approximately three months before the first administration of EMB-02.

The preliminary PK analysis included data from all available patients. EMB-02 demonstrated an approximately dose-proportional pattern across the dose range of 6 mg to 900 mg following the first dose, as shown in Fig. 3. The mean half-life was approximately 4 days, ranging from 2 to 5 days, at near steady state, supporting the once-weekly dosing schedule.

The preliminary immunogenicity assessment of EMB-02 indicates a relatively high incidence of immunogenicity, with 44 out of 46 (95.7%) evaluable patients showing positive results. The median onset for all evaluable patients who developed this condition was 14 days. The ADA titer exhibited an inverse trend relative to dose levels. At doses of 6 and 20 mg, some patients exhibited higher ADA titers, whereas at doses greater than 60 mg, the ADA titer was lower for the majority of patients, potentially indicating a low impact on PK exposure. In particular, the three patients who achieved CR status appeared to be ADA positive. There appeared to be no difference in ADA incidence between patients with best response of progressive disease and those with clinical benefit (i.e., SD/CR), as 20 out of 21 patients in each response category showed positive ADA. However, the ADA titer had an impact on the occurrence of IRRs/hypersensitivity, as patients with post-treatment ADA readouts who experienced IRRs/hypersensitivity (*n* = 12) had higher maximum ADA titers compared to those who did not (*n* = 32). The impact of corticosteroid prophylaxis on ADA development remains to be further clarified due to current data limitations. Detailed titer of ADAs across different cycles for each patient and the relationship between ADA exposure IRR and efficacy were summarized in Supplementary Table S4.

PD-1 receptor occupancy on circulating CD3 + T cells in 30 patients were evaluated. As shown in Fig. 3b, dose of 360 mg or higher resulted in sustained saturation of PD-1 receptors on circulating CD3 + T cells, while dose of 60 mg achieved approximately 80% PD-1 receptor occupancy. The three patients who achieved CR status exhibited similar PD-1 receptor occupancy compared to other patients within the same cohort. Notably, two CR patients were from 60 mg cohort, suggesting that near-maximal receptor occupancy may be sufficient to drive meaningful antitumor activity. Based on integrated data on receptor occupancy, clinical efficacy, and patient safety, enrichment cohorts with doses of 60 mg, 180 mg, and 600 mg were initiated.

Forty-five pretreatment tumor specimens (37 archival, 8 freshly collected) were analyzed for PD-L1, LAG-3, and MHC II expression (Supplementary Table S5). Among the three patients who achieved CR, two exhibited moderate to strong expression of PD-L1, LAG-3, and MHC II. The third CR patient’s sample could not be evaluated due to suboptimal sample quality. Overall, the expression levels of PD-L1, LAG-3 and MHC II in tumors varied and no clear relationship between treatment response and expression levels was identified.

## Discussion

EMB-02 was designed as a dual immune checkpoint inhibitor capable of simultaneously binding to both PD-1 and LAG-3 on exhausted T cells, which typically exhibit deeper exhaustion with the co-expression of multiple inhibitory receptors [7]. Literature also suggests a significant correlation between LAG-3 expression on TIL and PD-1 [12]. This dual target design may enhance the synergistic blockade of these two immune checkpoints more effectively than using two separate antibodies, potentially leading to a more robust reactivation of exhausted T cells and improved antitumor immune response. Additionally, the bispecific design increases the likelihood that binding to one target will facilitate simultaneous engagement with the other. In preclinical studies, EMB-02 demonstrated an additive effect through dual-blockade of PD-1 and LAG-3-mediated inhibitory signals compared to treatment with anti-PD-1 or anti-LAG-3 monoclonal antibodies alone. EMB-02 also induced co-degradation of both PD-1 and LAG-3 on activated T cells, resulting in the complete depletion of PD-1, which cannot be achieved by either monoclonal antibodies alone or in combination.

In this phase I study, EMB-02 exhibited a favorable safety profile. All grade and grade 3 or higher TRAE rates were 68.1% and 12.8% respectively, comparable to other PD-1/LAG-3 bispecific antibodies and slightly higher than anti-PD-1/PD-L1 monoclonal antibodies alone [17, 20, 23]. In contrast, combined anti-LAG-3 and anti-PD-1 monoclonal antibody therapy may have a higher incidence of all grade and high grade events, as seen with the combination of nivolumab and relatimab, and fianlimab and cemiplimab [17, 24]. One DLT was observed in a patient treated at 900 mg, QW. According to the BOIN dose escalation rule, the MTD of EMB-02 was not reached; however, dose escalation was cautiously capped at 900 mg, QW.

Immune-mediated hepatic toxicity accounted for the most common high grade irAE (any grade: *n* = 3, 6.4%; grade≥3: *n* = 2, 4.3%) and is comparable to Opdualag and MGD-013 with reported rates ranging from 3.8 to 5.6% for any grade and 3.8–3.9% for high grade [17, 20]. All three patients experienced immune-mediated hepatotoxicity recovered with immunosuppressive therapy, although all discontinued EMB-02 due to the AE. Other immune related hepatic AEs related to EMB-02 included AST/ALT/blood bilirubin increased (6.4% each), while other compounds of same target showed relatively higher incidence of transaminitis than EMB-02 with a range of 7.3–28.9% for AST increased, 7.9–22.2% for ALT increased [21, 25, 26]. In general, these data indicate that EMB-02 has a similar immune related hepatotoxicity profile compared to other products with similar mechanism of action, although it is slightly higher than that observed with anti-PD-1 antibodies alone, likely reflecting the involvement of LAG-3 blockage in liver flare. These hepatic irAEs are generally manageable and reversible. Currently, the reason for the observed increased hepatotoxicity is not fully elucidated. However, one possible hypothesis is that the systemic introduction of anti-LAG-3 may block the innate immune inhibitory functions of FGL-1 and LSECtin, which are highly expressed in the liver [27, 28]. This disruption of immune tolerance could lead to a flare in immune response in the liver, resulting in immune cell infiltration and hepatitis [29]. Additionally, underlying disease in patients may also partially contribute to the immune mediated hepatotoxicity. A retrospective analysis has demonstrated that melanoma and liver metastasis are both risk factors for CPI induced hepatotoxicity [30], with three out of the four patients in this study who reported immune-mediated liver injury had melaoma, and two patients with high grade immune-mediated hepatitis had liver metastases. Furthermore, research suggests that endothelitis could be associated with anti-LAG-3-induced liver injury [31], indicating that the addition of LAG-3 may increase the risk of immune mediated liver injury. Although IRR was seen frequently (*n* = 11, 23.4%) and across all dose levels, almost all cases were low grade.With supportive treatments, most patients recovered on the same day and tolerated rechallenge with subsequent prophylaxis.

The overall efficacy of EMB-02 was modest, with an ORR and DCR of 6.4% and 44.7%, respectively, however generally comparable with other anti-PD-1 and anti-LAG-3 bispecific antibodies in dose escalation studies which did not for selected tumor type [20, 23]. In our study, the modest activity observed may be partly due to the tumor types enrolled, as a high proportion of patients had non CPI-sensitive tumor types, such as MSS CRC (*n* = 10, 21.3%) and non-cutaenous melanoma (*n* = 10, 21.3%, including one sybtype unknown) or had CPI-exposure in those with CPI-sensitive tumors. In general, EMB-02 demonstrated better efficacy in CPI naïve patients, consistent with other LAG-3-targeting strategies [17, 20, 24].

Turning to cutaneous melanoma, a large proportion of patients have already received anti-PD-1 treatment in adjuvant or neoadjuvant setting [24], or have developed primary and/or secondary resistance in the advanced setting, and treatment with the comination of ipilimumab and nivolumab is associated with higher toxicity [32]. Therefore, there is an urgent need for new CPI combinations which are effective and safe in this population. In this study, two cutaneous melanoma with prior CPI treatment gained durable clinical benefit, highlighting the potential value of EMB-02 in this population.

For CRC, all ten patients enrolled in the study are MSS and PD-L1 low expression with median 3 lines of prior therapy. Notably, no responses were seen, while durable SDs were observed. A Phase 3 trial (RELATIVITY-123), investigating Opdualag in patients with previously treated metastatic MSS CRC, was recently terminated due to futility, as it was not expected to meet its primary end point based on a planned analysis by an independent data monitoring committee. Another combination of favezelimab (anti-LAG-3) plus pembrolizumab in 80 MSS CRC patients in a phase 1 trial reported an overall ORR of 6.3% (5/80), with a higher response rate (11% [4/36]) in the PD-L1 CPS ≥ 1 population [33]. A phase 3 active control trial is currently ongoing in PD-L1 positive CRC [34]. Thus far, immunotherapies have demonstrated limited efficacy in MSS CRC, emphasizing the need for more treatment options.

Prior studies have shown a high percentage of T cells in tumor-infiltrating lymphocytes (TILs) in ESCC tumors, and LAG-3 expression may be high on CD8 + TIL from ESCC, playing an important role in regulating CD8 TIL function alongside PD-1. ESCC may also have high MHC-II expression, which can act as a ligand to induce the LAG-3 inhibitory signal [35–37]. LBL-007, an anti-LAG-3 monoclonal antibody, also showed preliminary activity in ESCC in an early phase clinical study, with partial response or tumur reduction in three of five treated CPI naïve patients [38]. In the present study, three of three patients with ESCC, all CPI-naïve and with relatively high PD-L1 and LAG-3 expression derived durable clinical benefit.

No clear relationship was observed between EMB-02 activity and tumoral expression levels of PD-L1, LAG-3, or MHC II. This lack of correlation may be attributed to several confounding factors, including diverse tumor types, varying EMB-02 dose levels, and limited sample size. To further elucidate potential predictive factors of EMB-02 efficacy, future investigations could focus on alternative biomarkers such as tumor mutational burden (TMB), immune gene expression profiles, single-cell sequencing, genomic, and proteomic analyses or other indicators of tumor microenvironment and immune dynamics.

PK analysis indicated that EMB-02 has a half-life of approximately 4 days, supporting a once-weekly dosing schedule. Although, EMB-02 exhibited a relatively high incidence of immunogenicity, there was minimal impact on PK exposure at higher dose levels. Further exploration of these relationships through pharmacokinetic and pharmacodynamic modeling, coupled with observed response rates and safety data, could provide a rationale for studying doses ranging from 180 to 600 mg in subsequent clinical trials [39].

Overall, the encouraging safety profile and early efficacy signals of EMB-02 supports its further development. Given the limited sample size, hetereogeneous tumor types and heavily pre-treated patients in this study, further research is need to decipher the optimal subpopulations who could benefit from this strategy. ESCC emerges as a potential direction for development for EMB-02, either as monotherapy or in combination with other agents. Meanwhile second line or third line cutaneous melanoma post CPI treatment is a possible direction as well. Additionally, future well-designed studies may address the challenges of determining the additional role of LAG-3 targeting to PD-1 inhibition in specific disease settings, and the comparative effectiveness of bispecific antibody versus combined monoclonal antibodies.

In conclusion, EMB-02 demonstrated a favorable safety profile and showed early efficacy signals in patients with advanced solid tumors. A higher clinical benefit rate was observed in CPI naïve patients, compared to those CPI-treated patients, although 2 out of 2 CPI-treated cutaneous melanoma patients showed durable clinical benefit in this study. This work preliminarily demonstrated the value of EMB-02 in advanced solid tumors, warranting its further development.

## Supplementary information

Supplementary tables

## Data Availability

Data is available within the manuscript and supplementary information.
