# Supplementary material for: First-in-human phase I study of EMB-02, a bispecific antibody targeting PD-1 and LAG-3 in patients with advanced solid tumors
Source: Br J Cancer. 2025 Apr 15;132(10):905–12. doi: 10.1038/s41416-025-02990-x (PMC12081902; doi:10.1038/s41416-025-02990-x)
Supplement: Supplementary file 1 — Supplementary tables [file 41416_2025_2990_MOESM1_ESM.docx]

**First-in-human phase I study of EMB-02, a bispecific antibody targeting PD-1 and LAG-3 in patients with advanced solid tumors**

**Supplementary**

**Table S1 Treatment emergent adverse events (TEAEs) with an incidence of ≥10% in the safety population**

|  | **Total (N = 47)** | |
| --- | --- | --- |
| **Preferred Term** | **Any grade**  **n (%)** | **Grade ≥3**  **n (%)** |
| **Subjects with Any Treatment-emergent AE (%)** | 46 ( 97.9) | 23 ( 48.9) |
| **Fatigue** | 13 ( 27.7) | 0 |
| **Nausea** | 13 ( 27.7) | 0 |
| **Infusion related reaction** | 11 ( 23.4) | 2 ( 4.3) |
| **Vomiting** | 11 ( 23.4) | 0 |
| **Constipation** | 10 ( 21.3) | 0 |
| **Anaemia** | 9 ( 19.1) | 5 ( 10.6) |
| **Diarrhoea** | 9 ( 19.1) | 0 |
| **Cough** | 8 ( 17.0) | 0 |
| **Decreased appetite** | 8 ( 17.0) | 0 |
| **Blood LDH increased** | 7 ( 14.9) | 0 |
| **Pyrexia** | 7 ( 14.9) | 1 ( 2.1) |
| **ALT increased** | 6 ( 12.8) | 2 ( 4.3) |
| **Arthralgia** | 6 ( 12.8) | 0 |
| **GGT increased** | 6 ( 12.8) | 2 ( 4.3) |
| **Oedema peripheral** | 6 ( 12.8) | 1 ( 2.1) |
| **Urinary tract infection** | 6 ( 12.8) | 0 |
| **AST increased** | 5 ( 10.6) | 1 ( 2.1) |
| **Dehydration** | 5 ( 10.6) | 0 |
| **Pruritus** | 5 ( 10.6) | 0 |
| **Weight decreased** | 5 ( 10.6) | 0 |

GGT: Gamma-glutamyltransferase; ALT: Alanine aminotransferase ; AST: Aspartate aminotransferase ; LDH: lactate dehydrogenase

**Table S2 Immune-related AEs (irAEs) of any grade reported by at least 2 patients**

|  | **6mg QW (N = 5)** | | **20mg QW (N = 3)** | | **60mg QW (N = 11)** | | **180mg QW (N = 10)** | | **360mg QW (N = 5)** | | **600mg QW (N = 8)** | | **900mg QW (N = 5)** | | **Total (N = 47)** | | |
| --- | --- | --- | --- | --- | --- | --- | --- | --- | --- | --- | --- | --- | --- | --- | --- | --- | --- |
| **Preferred Term** | **Any grade**  **n (%)** | **Grade ≥3**  **n (%)** | **Any grade**  **n (%)** | **Grade ≥3**  **n (%)** | **Any grade**  **n (%)** | **Grade ≥3**  **n (%)** | **Any grade**  **n (%)** | **Grade ≥3**  **n (%)** | **Any grade**  **n (%)** | **Grade ≥3**  **n (%)** | **Any grade**  **n (%)** | **Grade ≥3**  **n (%)** | **Any grade**  **n (%)** | **Grade ≥3**  **n (%)** | **Any grade**  **n (%)** | **Grade ≥3**  **n (%)** |  |
| **Subjets with Any Drug-related AE (%)** | 0 | 0 | 0 | 0 | 2 ( 18.2) | 0 | 5 ( 50.0) | 1 ( 10.0) | 0 | 0 | 4 ( 50.0) | 1 ( 12.5) | 3 ( 60.0) | 3 ( 60.0) | 14(29.8) | 5 ( 10.6) |  |
| **Immune mediated hepatitis** | 0 | 0 | 0 | 0 | 0 | 0 | 1 ( 10.0) | 1 ( 10.0) | 0 | 0 | 0 | 0 | 2 ( 40.0) | 1 ( 10.0) | 3 ( 6.4) | 2 ( 4.3) |  |
| **Hypothyroidism** | 0 | 0 | 0 | 0 | 0 | 0 | 2 ( 20.0) | 0 | 0 | 0 | 1 ( 12.5) | 0 | 0 | 0 | 3 ( 6.4) | 0 |  |
| **Pruritus** | 0 | 0 | 0 | 0 | 0 | 0 | 1 ( 10.0) |  | 0 | 0 | 2 ( 25.0) | 0 | 0 | 0 | 3 ( 6.4) | 0 |  |
| **ALT increased** | 0 | 0 | 0 | 0 | 0 | 0 | 0 | 0 | 0 | 0 | 1 ( 12.5) | 1 ( 12.5) | 1 ( 20.0) | 1 ( 20.0) | 2 ( 4.3) | 2 ( 4.3) |  |
| **AST increased** | 0 | 0 | 0 | 0 | 0 | 0 | 0 | 0 | 0 | 0 | 1 ( 12.5) | 0 | 1 ( 20.0) | 1 ( 20.0) | 2 ( 4.3) | 1 ( 2.1) |  |
| **GGT increased** | 0 | 0 | 0 | 0 | 0 | 0 | 0 | 0 | 0 | 0 | 1 ( 12.5) | 1 ( 12.5) | 1 ( 20.0) | 1 ( 20.0) | 2 ( 4.3) | 2 ( 4.3) |  |
| **Lipase increased** | 0 | 0 | 0 | 0 | 0 | 0 | 0 | 0 | 0 | 0 | 0 | 0 | 2 ( 40.0) | 2 ( 40.0) | 2 ( 4.3) | 2 ( 4.3) |  |
| **Hyperthyroidism** | 0 | 0 | 0 | 0 | 0 | 0 | 0 | 0 | 0 | 0 | 2 ( 25.0) | 0 | 0 | 0 | 2 ( 4.3) | 0 |  |
| **Pyrexia** | 0 | 0 | 0 | 0 | 0 | 0 | 1 ( 10.0) | 0 | 0 | 0 | 1 ( 12.5) | 0 | 0 | 0 | 2 ( 4.3) | 0 |  |
| **Arthralgia** | 0 | 0 | 0 | 0 | 0 | 0 | 2 ( 20.0) | 0 | 0 | 0 | 0 | 0 | 0 | 0 | 2 ( 4.3) | 0 |  |

**Table S3 IRR symptoms of any grade reported by at least 2 patients**

|  | **6mg QW (N = 5)** | | **20mg QW (N = 3)** | | **60mg QW (N = 11)** | | **180mg QW (N = 10)** | | **360mg QW (N = 5)** | | **600mg QW (N = 8)** | | **900mg QW (N = 5)** | | **Total (N = 47)** | | |
| --- | --- | --- | --- | --- | --- | --- | --- | --- | --- | --- | --- | --- | --- | --- | --- | --- | --- |
| **Preferred Term** | **Any grade**  **n (%)** | **Grade ≥3**  **n (%)** | **Any grade**  **n (%)** | **Grade ≥3**  **n (%)** | **Any grade**  **n (%)** | **Grade ≥3**  **n (%)** | **Any grade**  **n (%)** | **Grade ≥3**  **n (%)** | **Any grade**  **n (%)** | **Grade ≥3**  **n (%)** | **Any grade**  **n (%)** | **Grade ≥3**  **n (%)** | **Any grade**  **n (%)** | **Grade ≥3**  **n (%)** | **Any grade**  **n (%)** | **Grade ≥3**  **n (%)** |  |
| **Chills** | 0 | 0 | 1 ( 33.3) | 0 | 1 ( 9.1) | 0 | 2 ( 20.0) | 0 | 0 | 0 | 3 ( 37.5) | 0 | 0 | 0 | 7 ( 14.9) | 0 |  |
| **Nausea** | 1 ( 20.0) | 0 | 0 | 0 | 1 ( 9.1) | 0 | 1 ( 10.0) | 0 | 0 | 0 | 1 ( 12.5) | 0 | 0 | 0 | 4 ( 8.5) | 0 |  |
| **Back pain** | 0 | 0 | 0 | 0 | 0 | 0 | 1 ( 10.0) | 0 | 0 | 0 | 1 ( 12.5) | 0 | 1 ( 20.0) | 0 | 3 ( 6.4) | 0 |  |
| **Pruritus** | 0 | 0 | 1 ( 33.3) | 0 | 1 ( 9.1) | 0 | 1 ( 10.0) | 0 | 0 | 0 | 0 | 0 | 0 | 0 | 3 ( 6.4) | 0 |  |
| **Abdominal pain** | 0 | 0 | 0 | 0 | 0 | 0 | 1 ( 10.0) | 0 | 0 | 0 | 1 ( 12.5) | 0 | 0 | 0 | 2 ( 4.3) | 0 |  |
| **Pyrexia** | 0 | 0 | 0 | 0 | 0 | 0 | 1 ( 10.0) | 0 | 0 | 0 | 1 ( 12.5) | 0 | 0 | 0 | 2 ( 4.3) | 0 |  |
| **Urticaria** | 0 | 0 | 1 ( 33.3) | 0 | 1 ( 9.1) | 0 | 0 | 0 | 0 | 0 | 0 | 0 | 0 | 0 | 2 ( 4.3) | 0 |  |
| **Dyspnoea** | 0 | 0 | 1 ( 33.3) | 0 | 0 | 0 | 0 | 0 | 0 | 0 | 0 | 0 | 1 ( 20.0) | 0 | 2 ( 4.3) | 0 |  |

**Table S4 EMB-02 immunogenicity titer summary by individual patient**

| Cohort | Subject | BOR^d^ | IRR/Hypersensitivity^c^ | Schedule Visit^a^ | | | | | | | | | | | |
| --- | --- | --- | --- | --- | --- | --- | --- | --- | --- | --- | --- | --- | --- | --- | --- |
|  |  |  |  | C1D1 | C1D15 | C2D1 | C3D1 | C5D1 | C7D1 | C9D1 | C11D1 | C13D1 | C15D1 | EOT | EOT 30days |
|  |  |  |  | ADA Titer | | | | | | | | | | | |
| 6mg | #1 | SD | None | Neg | 133.63 | 773.27 |  | 1567.45 | 3060.05 |  |  |  |  | 1404.42 |  |
|  | #2 | CR | None | Neg | 15.52 | 94.2 | 7.76 | 8.77 | 15.87 |  | 5.34 | 9.23 | 7.71 |  |  |
|  | #3 | PD | None | Neg | 54.88 | 46.7 | 2845.44 |  |  |  |  |  |  | 9704.37 | 7236.27 |
|  | #4 | SD | None | Neg | 22.23 | 11.74 | 13.57 | 33.84 |  | 1.78 | Neg |  | Neg | Pos^b^ | Neg |
|  | #5 | PD | IRR | 8.31 | 459.78 |  |  |  |  |  |  |  |  |  |  |
| 20mg | #1 | SD | IRR | Neg | 49.89 |  | 1433.6 |  |  |  |  |  |  | 1985.39 |  |
|  | #2 | PD | None | Neg | Neg | 5.51 |  |  |  |  |  |  |  | 1.68 | 4.23 |
|  | #3 | PD | IRR | |  | 3038.06 |  |  |  |  |  |  |  | 3636.48 |  |
| 60mg | #1 | SD | None | Neg | Neg | Neg | Neg | 346.55 | 389.78 |  | 111.26 |  |  | 55.89 | 27.4 |
|  | #2 | CR | None | Neg | 129.28 | 120.83 | 243.48 | 213.1 | 215.51 | 127.63 |  |  |  |  |  |
|  | #3 | CR | None | Neg | 190.98 | 51.66 | 4.35 | 88.14 | 88.25 |  | 19.86 |  |  |  |  |
|  | #4 | PD | None | Neg | Pos^b^ | Neg |  |  |  |  |  |  |  |  |  |
|  | #5 | SD | None | Neg | 2.23 | Neg | Neg | 99.84 |  |  |  |  |  | 395.91 |  |
|  | #6 | PD | None | Neg | 48.69 |  |  |  |  |  |  |  |  |  |  |
|  | #7 | SD | None | Neg | 1.84 | 14.58 | 4.39 |  |  |  |  |  |  | 11.74 |  |
|  | #8 | PD | None | Neg | 198.45 | 31.89 |  |  |  |  |  |  |  | 3.28 | Neg |
|  | #9 | SD | None | Neg | 6.09 | 3.55 |  |  |  |  |  |  |  | 2.4 | 2.09 |
|  | #10 | ND | IRR | Neg | 125.06 | 2151.77 |  |  |  |  |  |  |  | 2874.03 | 506.44 |
|  | #11 | SD | IRR | Neg | 144 |  |  | 227.69 | 342.86 |  |  |  |  |  |  |
| 180mg | #1 | ND | None | Neg | 245.08 |  |  |  |  |  |  |  |  |  |  |
|  | #2 | SD | None | Neg | Neg | Neg | Neg | 1.07 | 1.74 |  |  |  |  |  |  |
|  | #3 | SD | None | Neg | 58.04 | 31.25 | 14.18 | 172.8 |  |  |  |  |  |  |  |
|  | #4 | PD | None | Neg | 3.84 | 2.51 |  |  |  |  |  |  |  | 1.13 |  |
|  | #5 | PD | None | Neg | Neg | 3.65 | 108.42 | 107.25 | 70.09 |  |  |  |  | 29.2 |  |
|  | #6 | SD | None | Neg | 6.46 | Neg | Neg | Neg | Neg | Neg |  |  |  | 3.83 | Pos^b^ |
|  | #7 | PD | None | Neg | 3.47 | 25.3 |  |  |  |  |  |  |  | 13.61 |  |
|  | #8 | SD | IRR | Neg | 470.7 | 981.18 | 7104.38 | 7813.12 | 9961.47 |  |  |  |  | 57395.74 | 50216.96 |
|  | #9 | PD | IRR | Neg | 326.22 | 60.05 | 133.69 |  |  |  |  |  |  | 893.35 | 747.52 |
|  | #10 | SD | None | Neg |  | 247.72 |  |  |  |  |  |  |  | 63.76 |  |
| 360mg | #1 | SD | None | Neg | 12.95 | 13.59 | 73.22 | 136.89 | 45.01 | 123.95 | 96.64 | 45.7 |  | 7.97 |  |
|  | #2 | PD | None | Neg | 2.9 | Neg |  |  |  |  |  |  |  | Neg |  |
|  | #3 | PD | None | Neg | 31.85 |  |  |  |  |  |  |  |  |  |  |
|  | #4 | PD | None | Neg | Neg | Neg |  |  |  |  |  |  |  | Neg |  |
|  | #5 | PD | None | Neg | 51.66 |  |  |  |  |  |  |  |  |  |  |
| 600mg | #1 | PD | IRR | Neg | 98.72 | 103.68 |  |  |  |  |  |  |  | 29.11 |  |
|  | #2 | SD | None | Neg | 19.1 | 56.96 |  |  |  |  |  |  |  |  |  |
|  | #3 | SD | None | Neg | 51.56 | 7.94 | 1.86 |  | Neg |  |  |  |  | Neg | Neg |
|  | #4 | SD | IRR | Neg | 7.91 |  |  |  |  |  |  |  |  |  |  |
|  | #5 | PD | IRR | Neg | 274.86 | 450.84 |  |  |  |  |  |  |  | 6383.46 |  |
|  | #6 | PD | None | Neg | 95.49 | 38.91 |  |  |  |  |  |  |  | 48.26 | 13.27 |
|  | #7 | SD | None | Neg | Neg | Neg | Neg | Neg |  |  |  |  |  |  |  |
|  | #8 | PD | Hypersensitivity | 1.46 | 79.64 | 283.41 | 193.06 |  |  |  |  |  |  | 284.16 |  |
| 900mg | #1 | ND | None | Neg | 89.4 |  |  |  |  |  |  |  |  |  |  |
|  | #2 | PD | IRR | Neg | 84.48 | 142.24 |  |  |  |  |  |  |  |  | 5.34 |
|  | #3 | PD | None | Neg | 2.02 | Neg | Neg |  |  |  |  |  |  | Neg | Neg |
|  | #4 | PD | None | Neg | Neg |  |  |  |  |  |  |  |  | 186.43 | 26.09 |
|  | #5 | ND | None | Neg | 15.46 |  |  |  |  |  |  |  |  | 9.85 |  |

Note: BOR: best overall response; Neg: negative; ND: not done; Pos: positive

1. On C17D1, C19D1, C23D1, C25D1, and C27D1, only one patient (1001-1002 at 6 mg) had an ADA titer within the range of 2.23 to 14.02, which were not listed in the table. Additionally, a total of six unscheduled ADA samples, collected from five patients (dose range: 6-60 mg), with titers ranging from 3.62 to 320.85, were also not presented in the table. This is because the unscheduled ADA titers for each patient were within the range of their respect scheduled ADA titers, except for patient #2 from 60mg cohort, whose titer was 320.85 on C2D22, exceeding his individual upper limit.
2. These ADA positive samples provided only qualitative results, without quantitative readouts.
3. Eleven patients reported with term “IRR”, one patient reported “hypersensitivity” with similar signs and symptoms to IRR.
4. BOR regardless of confirmation is determined by the best timepoint response. ND represents no post-baseline tumor assessment at any time point.

**Table S5 Expression Levels of PD-L1, LAG-3, and MHC II in Patient Tumor Samples**

| Patient ID | Tumor Type | Dose (mg) | Best overall Response | PD-L1 expression  (% Tumor cells expressing PD-L1) | PD-L1 expression  (% of immune cells expressing PD-L1) | % of Immune Cells Present | LAG3 expression  (% of inflammatory cells expressing LAG3 in tumor stromal area) | MHC II expression  (% of inflammatory cells expressing MHC II in tumor stromal area) | MHC II expression  (% of tumor cells expressing MHC II) | MHC II expression  (MHC II average staining intensity in tumor cells) |
| --- | --- | --- | --- | --- | --- | --- | --- | --- | --- | --- |
| 1 | C-MEL | 60 | CR | 0% | 50% | 10% | 1% | 80% | 0% | 0 |
| 2 | A-MEL | 60 | SD | 0% | 25% | 5% | 30% | 100% | 95% | 3+ |
| 3 | C-MEL | 180 | SD | N.A | N.A | N.A | 5% | 20% | 50% | 2+ |
| 4 | A-MEL | 180 | SD | 70% | 5% | 10% | 90% | 90% | 40% | 3+ |
| 5 | A-MEL | 600 | SD | 0% | 0% | 5% | 10% | 95% | 95% | 3+ |
| 6 | MEL-UNK | 20 | PD | 0% | 30% | 5% | 30% | 90% | 30% | 2+ |
| 7 | M-MEL | 180 | PD | 30% | 70% | 75% | 20% | 99% | 80% | 3+ |
| 8 | A-MEL | 360 | PD | 0% | <1.0% | 10% | 10% | 95% | 5% | 3+ |
| 9 | A-MEL | 600 | PD | 0% | 5% | 5% | 1% | 90% | 20% | 2+ |
| 10 | M-MEL | 900 | PD | 0% | <100.0% | 1% | 5% | 1% | 5% | 1+ |
| 11 | U-MEL | 900 | NA | 0% | <1.0% | 20% | 0% | 100% | 95% | 1+ |
| 12 | CRC | 6 | SD | 0% | 5% | 10% | 3% | 70% | 10% | 3+ |
| 13 | CRC | 180 | SD | 0% | 1% | 5% | 3% | 80% | 0% | 0 |
| 14 | CRC | 180 | SD | 0% | 1% | 5% | 1% | 80% | 0% | 0 |
| 15 | CRC | 600 | SD | 0% | 0% | 1% | 0% | 95% | 10% | 3+ |
| 16 | CRC | 600 | SD | 0% | 0% | 10% | 0% | 85% | 45% | 2+ |
| 17 | CRC | 6 | PD | 1% | 10% | 5% | 1% | 90% | 4% | 2+ |
| 18 | CRC | 360 | PD | 0% | 10% | 10% | 0% | 60% | 0% | 0 |
| 19 | CRC | 600 | PD | 0% | 1% | 5% | 0% | 99% | 0% | 0 |
| 20 | CRC | 600 | PD | 1% | 50% | 20% | 30% | 70% | 90% | 3+ |
| 21 | CRC | 60 | NA | 0% | 1% | 5% | 5% | 80% | 0% | 0 |
| 22 | NSCLC | 20 | SD | 0% | 10% | 20% | 10% | 80% | 0% | 0 |
| 23 | NSCLC | 60 | SD | 0% | 60% | 5% | 5% | 80% | 10% | 1+ |
| 24 | NSCLC | 600 | SD | 5% | 20% | 40% | 20% | 60% | 0% | 0 |
| 25 | NSCLC | 180 | NA | 0% | 0% | 5% | 0% | 60% | 0% | 0 |
| 26 | ESCC | 6 | CR | 5% | 25% | 50% | 8% | 70% | 50% | 2+ |
| 27 | ESCC | 60 | SD | 25% | 30% | 50% | 3% | 90% | 0% | 0 |
| 28 | ESCC | 180 | SD | 0% | 50% | 30% | 20% | 80% | 0% | 0 |
| 29 | GC | 6 | SD | 5% | 20% | 20% | 5% | 40% | 50% | 3+ |
| 30 | GC | 900 | PD | 3% | 20% | 50% | 20% | 90% | 95% | 3+ |
| 31 | GC | 900 | NA | 0% | 20% | 80% | 15% | 95% | 95% | 3+ |
| 32 | TNBC | 360 | SD | 1% | 5% | 20% | 3% | 80% | 10% | 2+ |
| 33 | TNBC | 60 | PD | 10% | 20% | 5% | 1% | 90% | 80% | 3+ |
| 34 | TNBC | 600 | PD | 0% | 5% | 10% | 2% | 95% | 1% | 1+ |
| 35 | HNSCC | 60 | SD | 25% | 30% | 10% | 30% | 95% | 90% | 3+ |
| 36 | HNSCC | 360 | PD | 30% | 1% | 5% | 10% | 80% | 60% | 2+ |
| 37 | EAC | 60 | PD | 0% | 60% | 10% | 5% | 80% | 5% | 1+ |
| 38 | EAC | 180 | PD | 25% | 40% | 5% | 5% | 60% | 70% | 2+ |
| 39 | ASCC | 6 | PD | 15% | 15% | 30% | 10% | 70% | 100% | 3+ |
| 40 | ASCC | 20 | PD | 0% | <100.0% | 1% | 0% | 80% | 0% | 0 |
| 41 | ThyCa | 60 | SD | 60% | 10% | 10% | 50% | 80% | 60% | 3+ |
| 42 | ACCPG | 60 | PD | 0% | 10% | 5% | 1% | 99% | 0% | 0 |
| 43 | EC | 180 | PD | 0% | 20% | 20% | 5% | 100% | 10% | 1+ |
| 44 | RCC | 180 | PD | 0% | 0% | 1% | 1% | 70% | 100% | 2+ |
| 45 | LMS | 900 | PD | 0% | 0% | 0% | 2% | 95% | 0% | 0 |

Note: ACCPG=Adenoid Cystic Carcinoma of the parotid gland; ASCC=Anal squamous cell carcinoma (ASCC); CRC=Colorectal cancer; EAC=Oesophageal adenocarcinoma; EC=Endometrial Carcinoma; ESCC=Esophageal squamous cell carcinoma (ESCC); GC=Gastric cancer(GC); HNSCC=Head and neck squamous cell carcinoma (HNSCC); LMS=Leiomyosarcoma; C-MEL=Cutaneous Melanoma; A-MEL=Acral Melanoma; M-MEL= Mucosal Melanoma; U-MEL= Uveal Melanoma; MEL UNK= Melanoma of Unknown Subtype; NSCLC=Non-small cell lung cancer (NSCLC); RCC=Renal Cell Carcinoma; SCC UNK=Metastatic SCC of unknown primary; TNBC=Triple Negative Breast Cancer (TNBC); ThyCa=Thyroid Carcinoma
